# The Ataxia (*ax* ^J^) Mutation Causes Abnormal GABA_A_ Receptor Turnover in Mice

**DOI:** 10.1371/journal.pgen.1000631

**Published:** 2009-09-04

**Authors:** Corinna Lappe-Siefke, Sven Loebrich, Wulf Hevers, Oliver B. Waidmann, Michaela Schweizer, Susanne Fehr, Jean-Marc Fritschy, Ivan Dikic, Jens Eilers, Scott M. Wilson, Matthias Kneussel

**Affiliations:** 1 Zentrum für Molekulare Neurobiologie Hamburg, Universität Hamburg, Hamburg, Germany; 2 Carl-Ludwig-Institut für Physiologie, Universität Leipzig, Leipzig, Germany; 3 Institut für Biochemie II, Universität Frankfurt, Frankfurt, Germany; 4 Klinik für Innere Medizin 1, Schwerpunkt Gastroenterologie und Hepatologie, Universität Frankfurt, Frankfurt, Germany; 5 Institute of Pharmacology und Toxicology, University of Zurich, Zurich, Switzerland; 6 Department of Neurobiology, University of Alabama at Birmingham, Birmingham, Alabama, United States of America; The Jackson Laboratory, United States of America

## Abstract

Ataxia represents a pathological coordination failure that often involves functional disturbances in cerebellar circuits. Purkinje cells (PCs) characterize the only output neurons of the cerebellar cortex and critically participate in regulating motor coordination. Although different genetic mutations are known that cause ataxia, little is known about the underlying cellular mechanisms. Here we show that a mutated *ax* ^J^ gene locus, encoding the ubiquitin-specific protease 14 (Usp14), negatively influences synaptic receptor turnover. *Ax* ^J^ mouse mutants, characterized by cerebellar ataxia, display both increased GABA_A_ receptor (GABA_A_R) levels at PC surface membranes accompanied by enlarged IPSCs. Accordingly, we identify physical interaction of Usp14 and the GABA_A_R α1 subunit. Although other currently unknown changes might be involved, our data show that ubiquitin-dependent GABA_A_R turnover at cerebellar synapses contributes to *ax* ^J^-mediated behavioural impairment.

## Author Summary

Cerebellar ataxia describes a combination of motor symptoms and uncoordinated movements that originates from various hereditary and non-hereditary diseases. Although functional disturbances of cerebellar inhibitory output signals are thought to cause ataxia, the underlying molecular mechanisms are barely understood and medical treatment therefore remains difficult. We analysed a behavioural abnormality up to the molecular level in a mouse mutant (*ax* ^J^) representing a model for ataxia. The *ax* ^J^ mutation reduces the expression level of a ubiquitin protease (Usp14) leading to an abnormal turnover of neurotransmitter receptors. Despite other yet unknown changes in *ax* ^J^ mutants, our data show that intracellular protein turnover contributes to a motor behavioural syndrome.

## Introduction

A number of heterogeneous hereditary and non-hereditary disorders lead to ataxia characterized by coordination failures [1],[2],[3]. The spontaneous *ax* ^J^ mutation affects the locomotory system, causing an ataxic phenotype in mice [4]. The mutated gene encodes the deubiquitinating enzyme (DUB) Usp14 [5], a member of the ubiquitin-specific protease family [6],[7]. Due to insertion of an intracisternal A-particle into intron 5, expression levels of full-length Usp14 in brains of *ax* ^J^ mice are reduced to about 5% [5].

Usp14 catalyzes the hydrolysis of isopeptide bonds in ubiquitin-protein conjugates [8]. Upon alternative splicing of exon 4, two isoforms of Usp14 are generated. The full-length isoform contains an addition of 33 amino acids, required for proteasome binding. Accordingly, binding of Usp14 to the proteasome is thought to be necessary for efficient hydrolyse activity of Usp14 [9],[10]. *Ax* ^J^ mice display an exclusive downregulation of the full-length isoform, thereby representing a specific knockdown of the proteasome binding form of Usp14. Although the proteasome is likely to be involved in the neurological dysfunctions [9], Usp14 is unable to process polyubiquitin chains [11]. Since, its physiological substrate is thought to be mono- or oligoubiquitinated [5], rather than representing a polyubiquitinated protein destined for degradation at the proteasome, Usp14 may have several functions in ubiquitin-signaling pathways.

Ubiquitination is a key process in the regulation of synapse formation and function [6],[7],[12]. Following endocytosis, ubiquitinated receptors are sorted for lysosomal degradation, thereby preventing their recycling to the plasma membrane [13],[14],[15]. For instance, the surface expression of glycine receptors (GlyRs) depends on ubiquitination, suggesting an important role for this process in the regulation of synaptic receptor levels [16]. Moreover, ubiquitination of inhibitory GABA_A_ receptors (GABA_A_Rs) has recently been shown to be activity-dependent and to regulate synaptic GABA_A_R accumulation [17].

GABA_A_Rs mediate the majority of fast synaptic inhibition in the mammalian brain. In the cerebellum, 75% of all GABA_A_Rs contain the α1 subunit [18], whereas PCs exclusively express α1-containing GABA_A_Rs [19],[20],[21].

PCs transform excitatory afferent signals to inhibitory efferents that target the neurons of the deep cerebellar nuclei (DCN) and vestibular nuclei (VN) [1],[22],[23]. Their inhibitory influence on DCN and VN neurons is a prerequisite for normal motor coordination, and even minor disturbances of cerebellar inhibition has been shown to cause uncoordinated movements and ataxia [1]. Hence, mouse mutants characterized by Purkinje cell degeneration, such as *pcd* or leaner mice suffer from ataxia [24],[25],[26],[27].

Here, we show that the downregulation of Usp14 in *ax* ^J^ mice is accompanied by a marked redistribution of intracellular α1-containing GABA_A_Rs to PC surface membranes, leading to enlarged IPSC amplitudes. We further demonstrate physical interaction of Usp14 and GABA_A_R α1, suggesting that Usp14 directly participates in the regulation of synaptic GABA_A_R turnover. Consistently, interference with GABA_A_R-Usp14 binding in a heterologous system mimics the *in vivo* observations. Our data demonstrate a new concept with the ubiquitin-proteasome system (UPS) representing a key player in synaptic neurotransmitter receptor regulation.

## Results/Discussion

### Reduced expression of Usp14 alters GABA_A_R signal intensities *in vivo*

**Figure 1:**
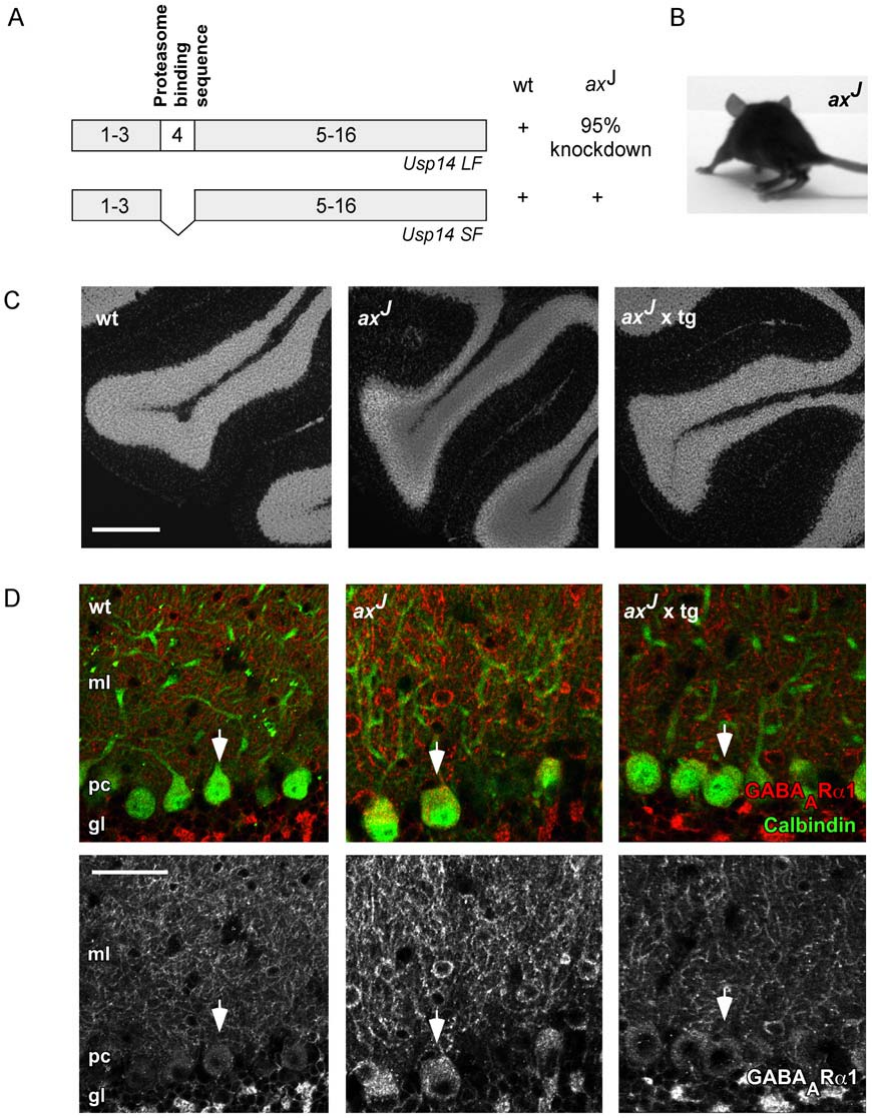
*Ax* ^J^ mice display altered *in vivo* surface membrane expression of α1-containing GABA_A_Rs. (A) Domain structure of the full-length long form (LF) and short form (SF) of *Usp14*. The *ax* ^J^ mutation leads to reduced expression of the Usp14LF [5]. (B) *Ax* ^J^ mice display ataxia and hind limb impairments. (C) The overall morphology of DAPI-stained *ax* ^J^ cerebellar slices appears normal, as compared to slices derived from wt and *ax* ^J^ mice that overexpress a neuron-specific *Usp14* transgene (*ax* ^J^ x tg). Scale bar: 500 µm. (D) Upper panels: Coimmunohistochemistry on cerebellar slices using calbindin-specific (green) antibodies to label Purkinje cells (arrows) and GABA_A_R α1 (red) specific antibodies. For a better visualization of the GABA_A_R α1 signal, the lower panels exclusively depict the red channel in black and white mode. *Ax* ^J^ mice display an increase of GABA_A_R α1 clusters especially in the molecular layer. Neuronal overexpression of a *Usp14* transgene on the *ax* ^J^ background (*ax* ^J^ x tg) rescues this effect *in vivo* (right). Scale bar: 100 µm. gl: granular layer; ml: molecular layer; pc: Purkinje cell layer.

Mice carrying the *ax* ^J^ mutation display reduced expression levels of the full-length Usp14 isoform in brain, whereas expression of the short Usp14 isoform remains unaltered ([5], Figure 1A). Phenotypically, *ax* ^J^ mice demonstrate severe coordination failures and ataxia (Figure 1B) [4], often linked to dysfunctions within cerebellar circuits [28]. Although increased apoptotic cell death is reported in *ax* ^J^-derived cerebellar granule cell layers [29], application of nuclear staining revealed that the overall architecture of the cerebellum remains normal (Figure 1C). To visualize PC bodies (arrows) and the molecular layer, representing PC dendrites, the PC marker protein Calbindin was immunolabeled. (Figure 1D, upper panels, green). Parallel staining of GABA_A_Rs using antibodies specific for the α1-subunit (Figure S1A-S1D) that represents the only α-type subunit in PCs, demonstrated strongly increased GABA_A_R cluster intensities in the molecular layer of *ax* ^J^ compared to wt cerebellum (Figure 1D, upper panels, red; lower panels, white). This phenomenon appeared to be mosaic and is in agreement with previous reports of variable expression levels of Usp14LF throughout different tissues [5]. Notably, these effects were specific and due to decreased Usp14 levels, since neuron-specific transgenic expression of Usp14 on the background of *ax* ^J^ mice [9] genetically reversed this effect, thereby leading to similar GABA_A_R α1 signal intensities as detected in wt PCs (Figure 1D, *ax* ^J^ x tg, right). Together, these data indicate that Usp14 regulates either the gene expression or the subcellular distribution of α1-containing GABA_A_Rs in PCs.

### Intracellular α1-containing GABA_A_Rs in PCs of *ax* ^J^ mice are redistributed to surface membranes

**Figure 2:**
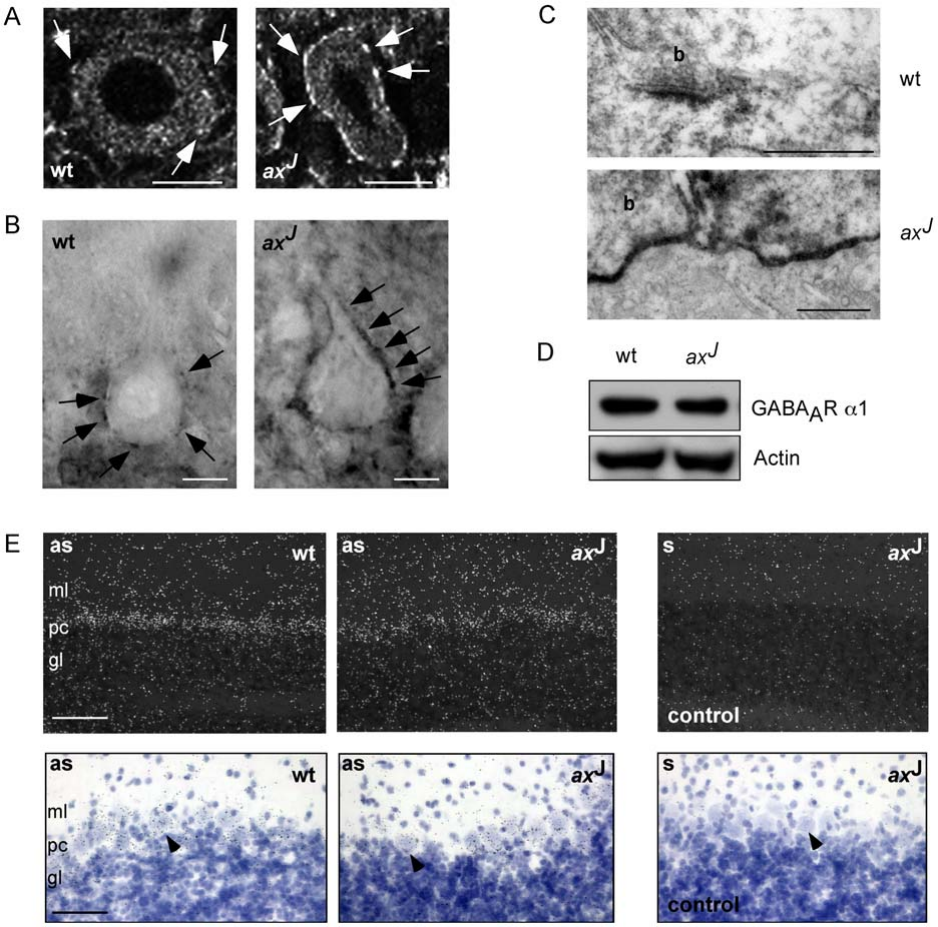
α1-containing GABA_A_Rs are redistributed in PCs of *ax* ^J^ mice. GABA_A_R α1 staining of PCs in slices of wt or *ax* ^J^ mice using Cy3- (A) or biotin- (B) labeled secondary antibodies. Scale bars: 20 µm. (C) EM analysis of GABA_A_R α1 using biotin-coupled secondary antibodies. Scale bars: 500 nm. (D) Western blot analysis of protein extracts from wt or *ax* ^J^ cerebella reveals that the effects seen in (A–C) represent a receptor redistribution rather than a change in total gene expression. Actin serves as a loading control. (E) *In situ* hybridisation using GABA_A_R α1 oligonucleotides revealed comparable mRNA levels in PC somata (arrows) as well as in dendrites of wt and *ax* ^J^ mice shown in dark (upper panels, white dots) and bright (lower panels, blue dots) field images. Nuclei were visualized by Hämalaun staining. Note that GABA_A_R α1 mRNA signals were absent upon use of sense control oligonucleotides (right panels). Scale bar, upper panel: 100 µm. Scale bar, lower panel: 50 µm. gl: granular layer; ml: molecular layer; pc: Purkinje cell layer.

To investigate the underlying mechanism of increased GABA_A_R clusters, PCs of wt and *ax* ^J^ mice were analyzed at the subcellular level. Immunostaining of GABA_A_R α1 using either fluorophore- (Figure 2A) or biotin-labeled (Figure 2B) secondary antibodies revealed a marked increase in GABA_A_R α1 clusters at the surface of cell bodies and proximal dendrites (Figure 2A and 2B, arrows). At the ultrastructural level, electron microscopy confirmed that large areas of the *ax* ^J^ PC surface, including extrasynaptic sites, were covered by α1-containing GABA_A_Rs (Figure 2C). Notably, the cytoplasm of PCs did not show increased signal intensities between the genotypes (Figure 2A–2C). In addition, western blot analysis of cerebellar protein extracts from wt and *ax* ^J^ mice (Figure 2D) as well as mRNA levels upon *in situ* hybridization (Figure 2E) demonstrated equal signals of GABA_A_R α1 proteins and mRNAs in both genotypes, indicating that the total gene expression of GABA_A_R α1 is not increased. We therefore conclude that a major loss of Usp14 expression leads to a surface redistribution of intracellular α1-containing GABA_A_Rs rather than to a significant increase in GABA_A_R α1 expression levels.

### Loss of Usp14 causes increased IPSC amplitudes in PCs

**Figure 3:**
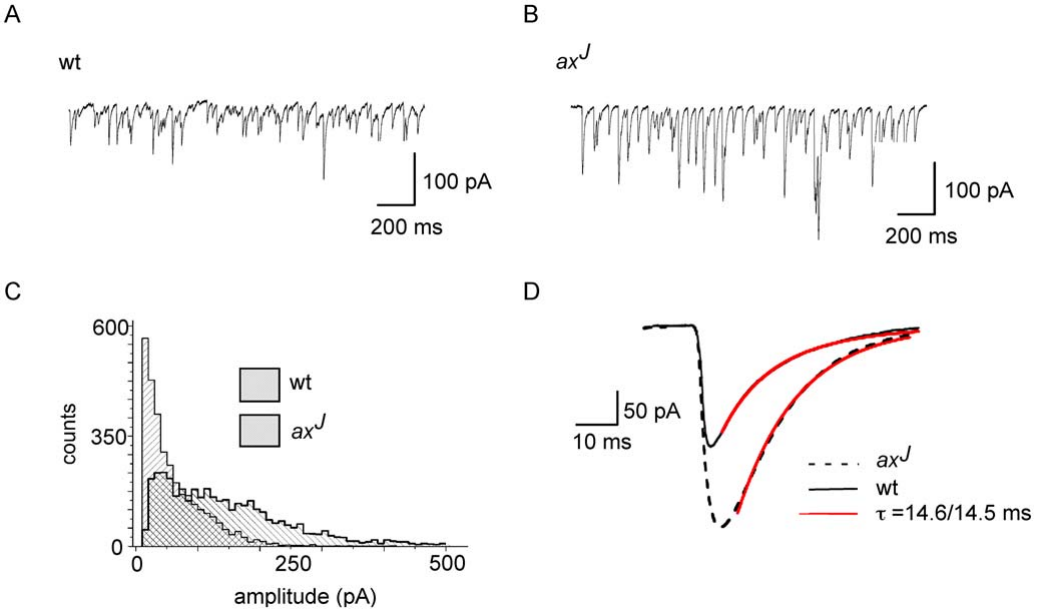
IPSC amplitudes of *ax* ^J^ PCs are reduced. (A,B) Traces of spontaneous IPSCs recorded from PCs of (A) wt or (B) *ax* ^J^ mice. Note that amplitudes in the *ax* ^J^ mutant are larger than in the wt. (C) For the given examples, IPSC amplitude distributions of wt and *ax* ^J^ PCs are shown. (D) The median value of IPSC amplitudes is 29±8 pA (n = 6) in wt, compared to 85±51 pA in *ax* ^J^ mice (n = 6).

Consistent with the immunohistochemical data, analysis of IPSCs (n = 10,000 events) indeed revealed that 67% of *ax* ^J^ PCs displayed a significant increase in GABAergic current amplitudes (Figure 3A–3D, and Figure S2A and Figure S2B). In parallel and as expected for a postsynaptic receptor phenomenon, the kinetic parameters, such as rise-time (10–90%) and decay-time (τ) remained unaltered under these conditions, indicating that both genotypes display no major changes in neurotransmitter uptake or release mechanisms (Figure S2C). However, the maximal amplitudes in *ax* ^J^ animals (>150 pA) displayed significantly (p = 0.005) higher decay time constants (τ = 12.1±3.1 ms), as compared to the decay time constants (τ = 10.3±2.9 ms) of maximal IPSC amplitudes in wt animals (80–150 pA; Figure S2C). Such differences are consistent with increased perisynaptic receptor numbers and support the immunochemical and EM observations. We therefore conclude that increased GABA_A_R levels at PC plasma membranes induce increased cerebellar inhibition, that leads to reduced inhibitory output levels of PCs. Notably, also PC degeneration (*pcd*) mutant mice display a severe decrease of PC inhibitory output and develop ataxia [1],[27]. In addition, GAT1 deficient mice, represented through prolonged GABA actions, due to disturbed GABA reuptake, suffer from ataxia [30]. Hence, altered inhibitory input to PCs leads to similar behavioral consequences compared to the loss of cerebellar inhibitory output upon Purkinje cell degeneration. However, if the observations in the present study contributed to the ataxia phenotype of *ax* ^J^ mice, one should identify a molecular link between GABA_A_R turnover and Usp14-mediated pathways.

### Usp14 and GABA_A_R α1 represent direct binding partners *in vitro*

**Figure 4:**
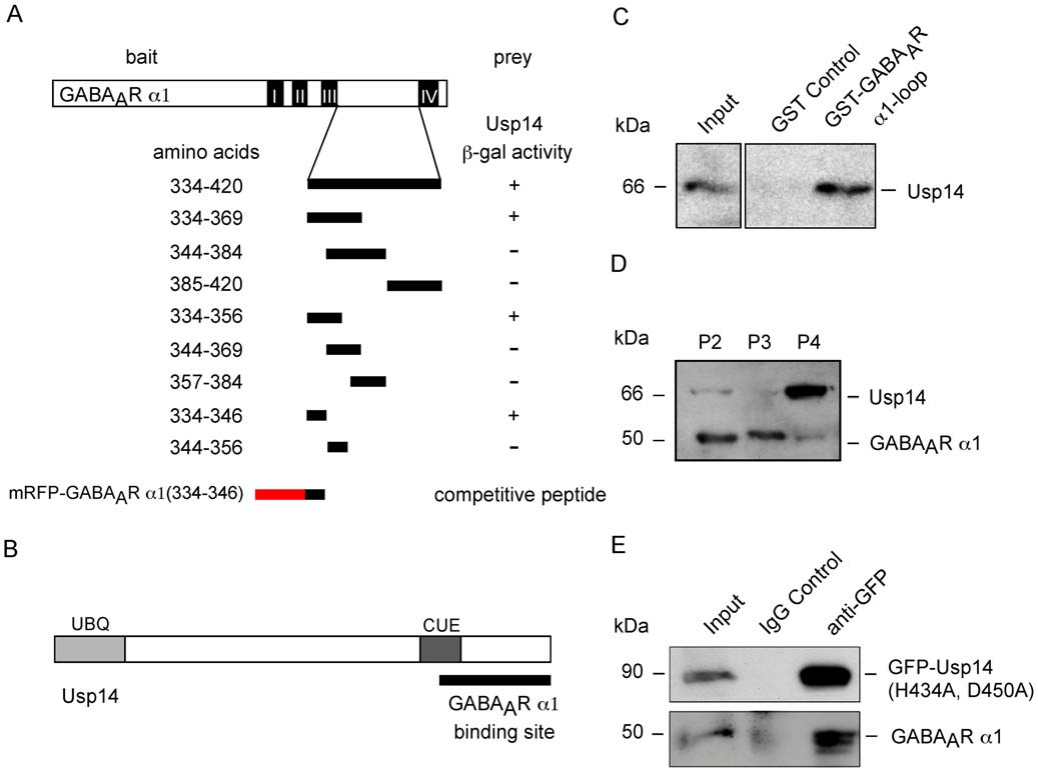
Usp14 and GABA_A_R α1 interact directly. (A) Domain structure of the GABA_A_R α1 subunit protein and mapping of the Usp14 binding site within its intracellular TM3-TM4 loop sequence, using the yeast two hybrid system. Peptide 334–346, derived from GABA_A_R α1, was fused to mRFP for the use as a competitive peptide (compare with Figure 6). (B) Domain structure of the Usp14 protein. GABA_A_R α1 binds to the Usp14 C-terminus. (C) Pulldown of endogenous Usp14 from brain extracts using a GST-tagged GABA_A_R α1 loop sequence. (D) GABA_A_R α1 and Usp14 cofractionate, but are enriched at non-overlapping peaks. (E) Coimmunoprecipitation of GABA_A_R α1 and a GFP-tagged Usp14 active site mutant (GFP-Usp14-H434A-D450A) upon expression in HEK293 cells.

To determine, whether Usp14 and GABA_A_R α1 might physically interact, we applied the lexA-based MATCHMAKER yeast two-hybrid system using Usp14LF (prey) and the large intracellular loop of GABA_A_R α1 (aa 334–420, bait). These experiments indeed revealed that Usp14 represents a direct GABA_A_R α1 binding partner (Figure 4A). Fine mapping, using systematic GABA_A_R α1 deletion mutants, identified the Usp14 binding region within the first 13 amino acids of the α1 loop sequence (aa 334–346) (Figure 4A). Vice versa, the GABA_A_R α1 binding site within the Usp14 protein was localized at its C-terminal domain (Figure 4B). In order to biochemically verify this interaction, we then applied a pull-down experiment using the GST-tagged GABA_A_R α1 loop (aa 334–420). Endogenous Usp14 protein derived from mouse brain lysates specifically bound to the immobilized GST-tagged GABA_A_R α1 loop, but not to GST alone (Figure 4C), indicating *in vitro* binding of the protease and the receptor polypeptide. Differential centrifugation of brain extracts revealed that both endogenous proteins cofractionate at P2 plasma-membrane (10,000×g), P3 vesicular (100,000×g), and P4 protein complex (400,000×g) fractions. However, while GABA_A_Rs are enriched at the plasma membrane (P2), Usp14 binds to the proteasome and is consequently enriched in fraction P4 (Figure 4D). This marginal overlap is consistent with a transient enzyme-substrate complex, however turned out not to be sufficient to obtain coimmunoprecipitation under standard conditions. Nevertheless, a GFP-tagged Usp14 mutant (GFP-Usp14(H434A-D450A)), harboring two point mutations within its functional catalytic domain [31], stabilized the complex, and enabled coprecipitation of both full-length binding partners derived from HEK293 cells (Figure 4E). Together, these data demonstrate physical interaction of GABA_A_R α1 and the ubiquitin-specific protease Usp14, and suggest that the observed GABA_A_R redistribution in ataxia mice (Figures 1 and 2) is directly caused by the loss of Usp14, thereby indicating that GABA_A_R turnover is ubiquitin-dependent.

### Usp14 and GABA_A_R α1 colocalize *in vitro* and *in vivo*

**Figure 5:**
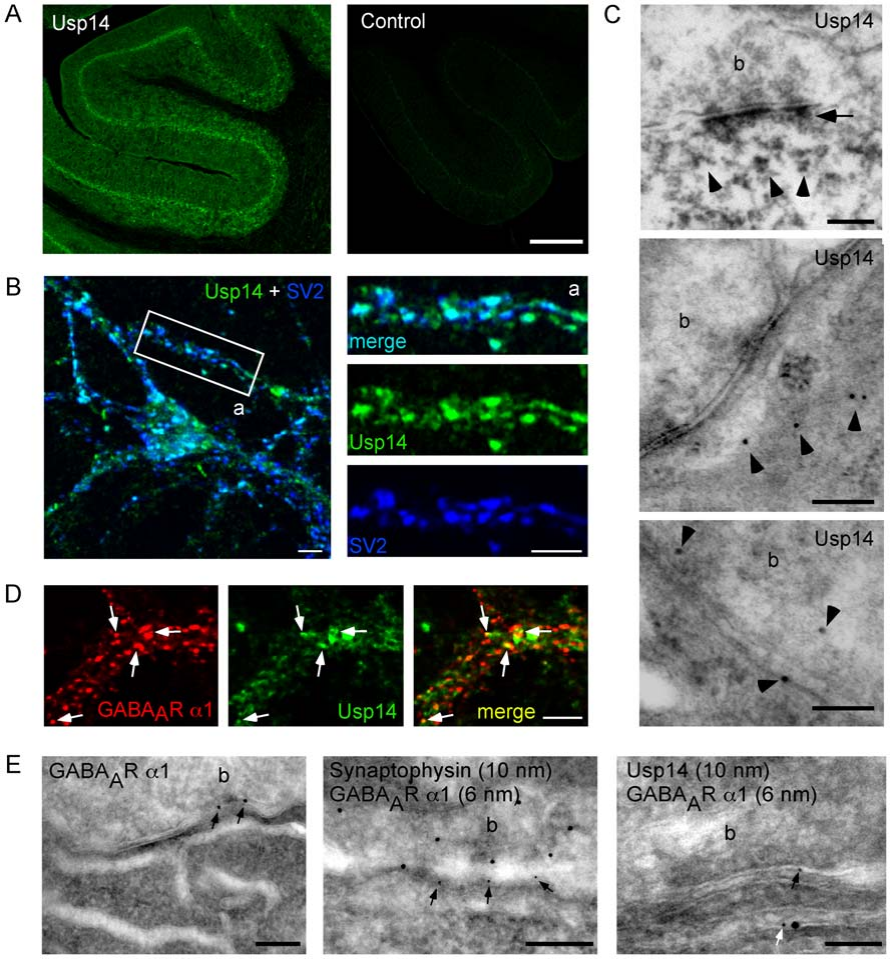
Usp14 and GABA_A_R α1 colocalize in cultured hippocampal neurons and cerebellar tissue slices. (A) Immunohistochemistry on brain slices using Usp14-specific antibodies (PB+2, left) or without primary antibody (control, right). Further specificity controls are described by Crimmins et al. [9]. Scale bar 350 µm. (B) Coimmunostaining of cultured hippocampal neurons using Usp14- (PB+2, green) and SV2- (blue) specific antibodies. Turquoise puncta indicate partial colocalization of Usp14 and SV2 as a synaptic marker protein. Scale bars: 5 µm. (C) Electron microscopy analysis of DAB- (upper panel) or gold-labeled (middle and lower panel) Usp14 protein in Purkinje cells of mouse cerebellar slices using Usp14-specific antibodies (PB+2). Scale bars: 200 nm. (D) Coimmunocytochemistry of Usp14 [10] (green) and GABA_A_R α1 (red) reveals partial colocalization in neurites of cultured hippocampal neurons (yellow puncta, arrows). Scale bar: 5 µm. (E) Electron microscopy upon single-immunogold-labeling using antibodies specific for GABA_A_R α1 (left) or double-immunogold-labeling of GABA_A_R α1 with either synaptophysin- (middle) or Usp14 (PB+2, right). GABA_A_R α1 signals are found in close proximity to Usp14 signals at tubular submembrane organelles (b; synaptic bouton) of mouse Purkinje cells (PCs). Scale bars, left and middle: 200 nm. Scale bar, right: 100 nm.

We next asked whether both proteins colocalize at the subcellular level. While GABA_A_R α1 subunits have been extensively characterized in both tissues and cells [32],[33], immunohistochemical analysis of Usp14 displayed a wide distribution in all layers of the cerebellum (Figure 5A) and was detected at synaptic vesicle protein 2 (SV2)-positive synaptic sites in both cultured hippocampal and cerebellar neurons (Figure 5B, and Figure S1E, turquoise). For analysis at ultrastructural resolutions, we performed immunoelectron microscopy using biotin- (Figure 5C, upper panel) or gold-labeled (Figure 5C, middle and lower panels) secondary antibodies. In this assays Usp14 was detected in close proximity to and directly at postsynaptic sites (Figure 5C, upper panel, arrows), at both the pre- and post-synapse, as well as directly at synaptic plasma membranes (Figure 5C, middle and lower panels, arrowheads).

In addition and in consistence with the *in vitro* binding data, coimmunostaining with antibodies specific for Usp14 and the GABA_A_R α1 subunit revealed partial colocalization in both cultured hippocampal and cerebellar neurons (Figure 5D, and Figure S1F, yellow, white arrows). At ultrastructural levels, this could be confirmed using gold-labeled secondary antibodies of different particle sizes. In accordance to the literature [33], GABA_A_R α1 (black arrows) was localized opposed to unlabeled (Figure 5E, left, arrows) or synaptophysin-positive presynaptic boutons (Figure 5E, middle, arrows), while colabeling of Usp14 and GABA_A_R α1 was rather detected at submembrane tubular organelles (Figure 5E, right, white arrow), described in both dendritic shafts and spines [34]. In addition to the smooth endoplasmic reticulum (SER), tubular compartments are generated through merge of internalized vesicles and multivesicular body (MVB)-tubule complexes and serve as intracellular stores of material destined for recycling or degradation [34],[35],[36],[37]. Given the fact that organelles that mediate neurotransmitter receptor sorting are localized subsynaptically [34],[38] with ubiquitin serving as a signal for internalization [39],[40], both the observed in *vitro* binding (Figure 4) and colocalization data (Figure 5) suggest that Usp14 represents a direct regulator of GABA_A_R turnover.

### GABA_A_Rs are ubiquitinated

To investigate whether GABA_A_R α1 might be a putative substrate for Usp14, we examined whether this subunit could be ubiquitinated in cells. Thus, HEK293T cells were transfected with GFP-tagged GABA_A_R α1, GABA_A_R β3, HA-tagged ubiquitin and either Usp14 wildtype (wt) or a catalytic mutant of Usp14, respectively (Figure S4). Extracts of untransfected HEK293T cells served as controls. Upon immunoprecipitation, using anti-GFP antibodies, GABA_A_R α1-GFP was precipitated from extracts containing GABA_A_R α1-GFP (Figure S4A, lower panel). Upon the use of HA-antibodies ubiquitinated forms of GABA_A_R α1 could be detected in extracts from transfected but not untransfected HEK293T cells (Figure S4A, upper panel). The detection of ubiquitinated GABA_A_R α1 is in line with a recent publication that reported ubiquitinated GABA_A_R β subunits [17], suggesting that GABA_A_Rs in general are subject to ubiquitin conjugation. In particular the abundance of ubiquitinated GABA_A_R α1 forms between 75 and 100 kDa (Figure S4A, upper panel, asterisk) is slightly increased in the presence of the Usp14 catalytic mutant, represented by a more intensive blurred signal (see magnified image in Figure S4B). This observation suggests a stabilization of mono-/oligoubiquitinated GABA_A_R α1 polypeptides upon binding of a functionally inactive form of Usp14. Thus, Usp14 might represent a critical DUB to GABA_A_R α1. A balanced control of GABA_A_R ubiquitination and deubiquitination might therefore be an important determinant in regulating GABA_A_R surface expression in neurons.

### Interference with GABA_A_R α1-Usp14 binding *in vitro* mimics *in vivo* observations from *ax* ^J^ mice

**Figure 6:**
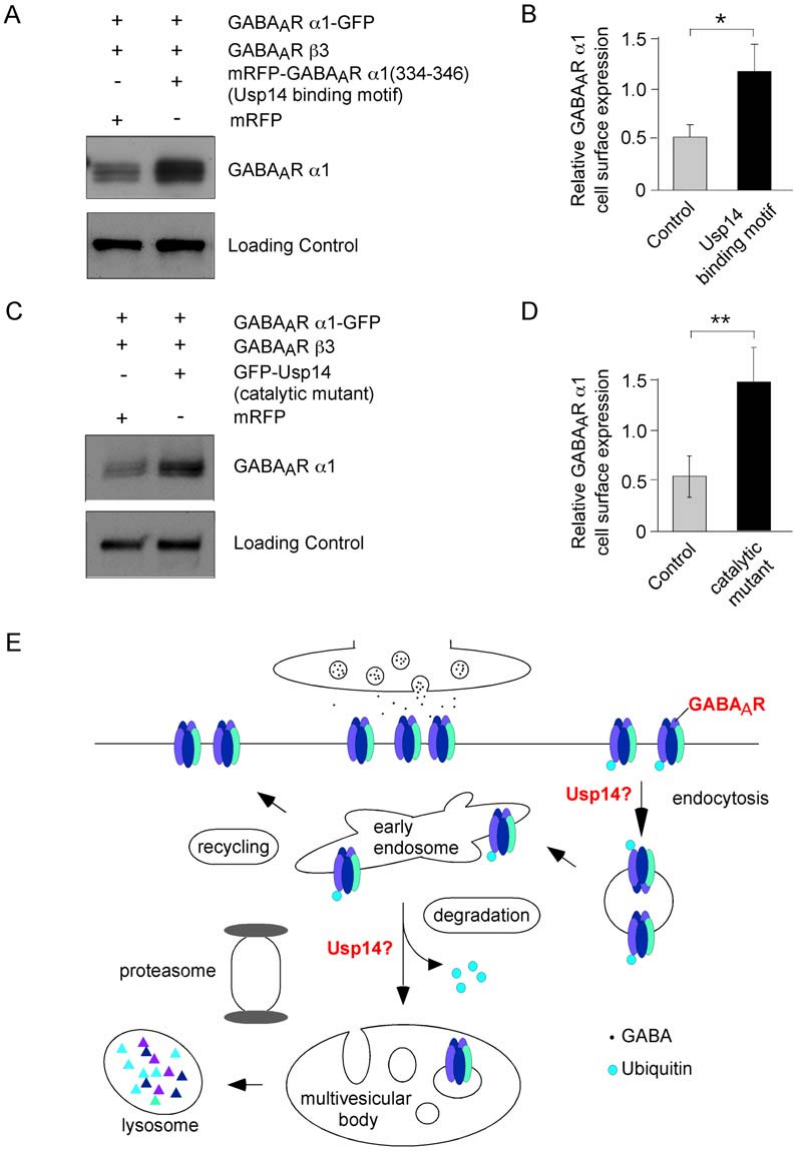
*In vitro* interference of GABA_A_R α1-Usp14 binding mimics *ax* ^J^ *in vivo* results. (A,B) Quantitative biotinylation assay upon HEK293 cell expression of GABA_A_R α1-GFP, GABA_A_R β3 with either mRFP or mRFP-GABA_A_R α1(334–346), respectively. GABA_A_R α1 cell surface expression is significantly increased through blockade of GABA_A_R α1–Usp14 binding in the presence of the competitive peptide mRFP-GABA_A_R α1(334–346). (C,D) Quantitative biotinylation assay upon HEK293 cell expression of GABA_A_R α1-GFP, GABA_A_R β3 with either mRFP or a catalytic Usp14 mutant, respectively. GABA_A_R α1 cell surface expression is significantly increased in the presence of inactive Usp14. Cadherin detection served as loading controls. (E) Model of GABA_A_R turnover and sorting of ubiquitinated receptors.

If the above interpretations were true, a minimal heterologous system should verify that Usp14 directly affects GABA_A_R turnover. In addition to the loss of Usp14 in mice, we aimed to proof, whether heterologous overexpression of an isolated Usp14 binding site (compare with Figure 4A) of GABA_A_R α1 would mimic the receptor surface distribution phenotype upon competitive interference with GABA_A_R α1–Usp14 binding. Thus, HEK293 cells were transfected with constructs encoding GABA_A_R α1-GFP, GABA_A_R β3 and the monomeric red fluorescent protein (mRFP)-tagged Usp14 binding site of GABA_A_R α1 (mRFP-GABA_A_R α1(334–346)) or with mRFP, respectively. Biotinylation of surface proteins, followed by immunoprecipitation, indeed revealed a 2.5-fold increase of GABA_A_R α1-GFP surface membrane levels in the presence of the competing peptide (Figure 6A and 6B), thereby leading to the same functional consequence, as observed in *ax* ^J^ mice. To verify both the expression and catalytical activity of Usp14 in HEK293 cells, we performed western blot analysis of protein extracts from kidney and cultured HEK293 cells, using a HA-tagged ubiquitin vinyl methyl ester (HAub-VME), active site probe [41],[42]. Western blot analysis using HA-specific antibodies confirmed that HEK293 cells express catalytically active Usp14 (Figure S3A). Hence, we conclude that the disruption of GABA_A_R α1-Usp14 binding, and consequently the gene expression knockdown of Usp14 in *ax* ^J^ mice, is directly causal for increased GABA_A_R surface membrane expression, known to result in enlarged IPSC amplitudes *in vivo*.

Since Usp14 represents a protease, its enzymatic activity should also be critical in this respect. To test this, we transfected HEK293 cells with constructs encoding GABA_A_R α1-GFP, GABA_A_R β3 and a catalytically inactive Usp14 mutant or with mRFP, respectively. Overexpression of the loss-of-function mutant, notably resulted in 3-fold enrichment of GABA_A_R α1-GFP surface expression (Figure 6C and 6D), as compared to control experiments, indicating that a balanced turnover of α1-containing GABA_A_Rs indeed requires a catalytically intact Usp14 enzyme.

In summary, these and other data in this study suggest that the function of Usp14 is directly involved in GABA_A_R turnover. Regulation of synaptic strength requires the precise control of neurotransmitter receptor numbers at synaptic sites. Our *in vivo* and *in vitro* observations in this study indicate the novel concept that DUB-dependent pathways regulate neurotransmitter receptor density and might participate in synaptic plasticity mechanisms. Since *ax* ^J^ mice represent mutants that are exclusively deficient for the proteasome-associated form of Usp14, with deubiquitinating enzymes playing an important role in the UPS, our data suggest a role of the proteasome in GABA_A_R turnover. It has been shown, that epidermal growth factor receptors (EGFRs), once activated, undergo ubiquitination and internalization from the plasma membrane [43]. Prior to their sorting into multivesicular bodies (MVBs), EGFRs require deubiquitination, a process that depends on proteasomal activity, although EGFRs, such as most transmembrane proteins, undergo degradation at lysosomes. Recent data in yeast further confirm a proteasomal contribution in similar processes, by showing that the proteasome-associated deubiquitinating enzyme Doa4 removes ubiquitin from cargo proteins prior to their entry into internal vesicles of MVBs [14],[44],[45].

Usp14, as it binds to the proteasome and mediates deubiquitination, is therefore a candidate factor to serve in similar pathways in neurons (Figure 6E). Consequently, Usp14 might represent the responsible DUB to trigger GABA_A_R transport from early endosomes into MVBs/lysosomes, although it might already interfere with GABA_A_Rs at the cell surface or right after internalization (Figure 6E). Interruption of this function, either by loss of Usp14 (*ax* ^J^ mice), or by interference with Usp14-GABA_A_Rs α1 binding (HEK293 cells), might either induce (i) backpropagation of disturbed endocytic pathways, leading to maintenance of the receptor at the cell surface or (ii) increased recycling of receptors back to the cell surface. Although an exact molecular mechanism remains to be elucidated, Usp14 represents a novel candidate to participate in the regulation of GABA_A_R turnover and synaptic plasticity at GABAergic synapses.

Since *ax* ^J^ animals show further neuronal abnormalities, such as impaired synaptic transmission at neuromuscular junctions, their neurological phenotype might be due to a combination of deficits. However, we conclude that impaired GABA_A_R turnover in PCs due to the loss of Usp14 significantly contributes to the ataxic phenotype in *ax* ^J^ mice. This view is supported by previous studies, which report that mouse mutants with altered GABA_A_R densities or reduced GABAergic terminals in the cerebellum, also develop severe motor impairments [1],[25],[27],[46]. For instance the *pcd* mouse mutant, characterized by a complete loss of PCs, shows ataxia. PC degeneration in *pcd* mice results in a loss of inhibitory PC output and consequently a reduced inhibitory input to the vestibular nuclei, representing one of the direct PC target regions. In addition, altered Purkinje cell input leads to ataxia. Hence, mice deficient for the GABA transporter GAT1 develop severe ataxia due to disturbed GABA re-uptake. Consequently, this functional deficit leads to an increased GABA_A_ receptor-mediated tonic conductance and prolonged IPSCs in both cerebellar granule and Purkinje cells [30]. It is therefore a possible scenario that motor impairments are closely linked to the level of inhibition within cerebellar circuits. The observed increase in GABA_A_R surface expression and IPSC amplitudes in *ax* ^J^ mice, as reported in this study, negatively affect PC functions and are likely to contribute to the ataxic symptoms. In parallel other yet unknown changes might be involved, too. Hence, in addition to GABAergic transmission, ubiquitin-mediated pathways might be putative targets for therapeutic treatment against certain forms of cerebellar ataxia.

## Methods

### DNA constructs

The entire cDNA sequence of GABA_A_R α1 was subcloned as an *EcoR*I/*Sal*I fragment into the pEGFP-N vector (BD Biosciences). The cDNA of Usp14 was subcloned as a *Bam*HI fragment into the pFLAG-CMV-2 vector (Sigma). HA-tagged Ubiquitin was subcloned as an *Eco*RI/*Xho*I fragment into the pcDNA3f vector (Invitrogen). Single mutations (Usp14: C114A; GABA_A_R α1 loop: introduction of stop codons to generate deletion mutants and the competitive peptide, respectively) or group mutations (Usp14: H434A-D450A) were introduced using the site-directed mutagenesis kit (Stratagene).

### Antibodies

Immunofluorescence: rabbit anti-GABA_A_R α1 (1∶250 Upstate); guinea pig anti-GABA_A_R α1 (1∶6000); rabbit anti-Usp14 [10] rabbit anti-Usp14 (138-R/PB+2, SM Wilson-lab.); mouse anti-Calbindin (1∶100, Sigma); mouse anti-synaptic vesicle (SV2, 1∶100, Hybridoma bank, University of Iowa); Secondary antibodies: CY3-, CY2- or CY5-conjugated donkey-anti rat, mouse, guinea pig or rabbit (all 1∶500, Dianova).

Immunoprecipitation/Western blot analysis: rabbit anti-GABA_A_R α1 (1∶500 Upstate, 1∶1000 AbD serotec); guinea pig anti-GABA_A_R α1 (1∶6000 JM Fritschy-lab.); mouse-anti-Usp14 (IA4; 1∶1000, SM Wilson-lab.) mouse anti-pan Cadherin (1∶100, Abcam); mouse anti-N-Cadherin (1∶4000, Cell Signaling Technology); rabbit anti-actin (1∶2000, Sigma); mouse anti-HA (1∶1000, Sigma; 1∶1000, Santa Cruz Biotechnology); anti-GFP (1∶1000, Roche), mouse anti-rpt4 (1∶1000, Biomol) Secondary antibodies: HRP-conjugated goat-anti rabbit, guinea pig and mouse (all 1∶10.000, Dianova); HRP-conjugated protein A (1∶1000, KPL); biotinylated secondary antibodies (1∶1000, Vector laboratories), gold-labeled secondary antibodies.

#### Transfection and immunostaining

Primary cultures of hippocampal or cerebellar neurons were prepared from mice (P0-P1) and cells were transfected using a Ca^2+^ phosphate coprecipitation protocol, as previously described (2.0–2.5 µg DNA/coverslip and 3.5 cm dish) [47],[48]. Immuno-histochemistry: wt and ax^J^ mice were anaesthetized and perfused with 2–4% paraformaldehyde (PFA). Brains were postfixed in 2–4% PFA for 1 h. For immunostaining floating vibratome sections (20 µm) were permeabilized (0,4% Triton X-100). After 30 min incubation in blocking solution (4% horse serum (HS), 0.2% TritonX-100) primary antibodies were applied (2% HS, 0.1% TrotinX-100). Secondary antibody incubation was performed for 2 h in 1.5% HS. Slices were analysed with an inverted Leica TCS-SP2 laser scanning confocal microscope (Leica). For double-labeling studies, a sequential scanning mode was used.

Immunocytochemistry: Primary neurons (DIV 12–14) were fixed in 4% PFA/4% sucrose (12 min) and washed in PBS prior to permeabilization with 0.25% Triton X-100 (4 min). Unspecific binding sites were blocked with 1% BSA for 30 min and cells were incubated with primary antibodies for 1 h at room temperature (RT). Cells were washed three times in PBS and incubated with secondary antibodies. Microscopy analysis was carried out with an inverted Leica TCS-SP2 laser scanning confocal microscope (Leica). For double-labeling studies, a sequential scanning mode was used.

### Electron microscopy

Preembedding immunocytochemistry: mice were anaesthetized and perfused with 4% PFA with 0.1% glutaraldehyde in PBS. Sagittal vibratome sections of the cerebellum were cut (60 µm). After washing in PBS, sections were treated with 0.3% H_2_O_2_ and 1% NaBH_4_ in PBS for 30 min. After rinsing in PBS, sections were incubated with 10% horse serum (HS) containing 0.2% BSA for 15 min and subsequently incubated over night with primary antibodies in PBS, containing 1% PS and 0.2% BSA (carrier). Sections were washed in PBS, incubated with biotinylated secondary antibody and diluted in carrier for 90 min. After rinsing, sections were incubated with ABC (Vector Labs) and diluted to a 1∶100 concentration in PBS for 90 min. Afterwards they were washed in PBS and further incubated in diaminobenzidine (DAB)-H_2_0_2_ solution (Sigma) for 10 min. Sections were then either mounted on glass coverslips (light microscopy) or postfixed with 1% OsO_4_, dehydrated in an ascending series of ethanol and embedded in Epon (Roth). Ultrathin sections were examined with a Zeiss EM 902.

Immunocytochemistry of ultrathin frozen sections: mice were perfused and cerebellar sections of the cerebellum were cut (100–200 µm), as described above. Small blocks of cerebellar tissue containing all layers were immersed in 12% gelatin in PBS at 37°C for 15–30 min. Blocks were transferred into vials containing 2.3 M sucrose in PBS and incubated over night. Thereafter they were frozen on specimen holders in liquid nitrogen. Ultrathin sections were prepared at a Reichard Ultracut microtome, equipped with a cryochamber and placed on copper grids (Sciences Services). Single and double immunogold labeling was performed according to Slot and Geuze using secondary 10 nm large protein A gold to label rabbit primary antibodies and 6 nm large gold (Dianova) to label guinea pig primary antibodies [49].

### Electrophysiology

Mice were anaesthetized and the cerebellar vermis prepared in ice-cold carboxygenated ACSF (NaCl 135 mM; KCl, 5 mM; CaCl_2_ 2 mM; MgCl_2_ 1 mM; glucose 10 mM; Na_2_HCO_3_, 30 mM; NaHPO_4_, 1.5 mM; pH 7.4 (bubbled with carbogen)). The tissue was cut into 200 µm sagittal sections (Microm HM 650V; Histoacryl glue, Braun), that were transferred to carboxygenated ACSF at 35°C for 20–30 min before being kept at RT (22–24°C), until further use.

Slices were placed in a recording chamber (RC26GLP, Warner Instr.) under an upright microscope (BX51WI, Olympus). Individual PCs were visually identified and recorded with borosilicate capillaries of approximately 5 MO resistance (Hilgenberg) using the whole-cell patch-clamp configuration. Spontaneous synaptic events were recorded under equimolare Cl^−^ concentrations at −60 mV and the GABAergic input isolated using the AMPA-type glutamate antagonist CNQX; the remaining IPSCs could be blocked by 20 µM bicuculline (Figure S2A). IPSC were recorded at 10 kHz for 60 s every 10 min. over approximately 1 h using the Patchmaster 2.05 software (HEKA). Data were analysed with the MiniAnalysis 6.02 program (Synaptosoft; converted with the supplied ABF Utility) using identical parameters for evaluating all IPSCs. Intracellular electrode: CsCl, 125 mM; MgCl_2_, 2 mM; EGTA 0.1 mM; TEA 5 mM; Na_2_-ATP, 4 mM; Na-GTP, 0.5 mM; HEPES, 10 mM; pH 7.3 (CsOH).

### Coimmunoprecipitation/GST-pulldown assay

HEK293 cells were harvested in 1% Triton X-100, 48 h after transfection. Antibodies were coupled to 30 µl of protein G beads (Dynal Biotech) in IP washing buffer (50 mM TrisHCl, 150 mM NaCl, 5 mM MgCl_2_, PH 7.1). Cell extracts were incubated with the beads over night, then washed and boiled in SDS sample buffer. For GST-pulldown experiments, HEK293 cells were harvested 48 h after transfection in 1 ml 1% Triton X-100. *E. coli* BL21 lysates were obtained by sonification and centrifugation at 10,000×g for 30 min. Bacterial lysates were coupled to glutathione-sepharose beads (Amersham) for 3 h. HEK293 cell lysates were applied to the beads for 10–12 h. Beads were washed and boiled prior to Western blot analysis. Proteins separated by SDS PAGE were transferred to PVDF or nitrocellulose membranes and unspecific binding sites were blocked using PBS containing 0.1% Tween and 5% skim milk powder. Primary and secondary antibody incubation was performed in blocking solution.

### Ubiquitination analysis

HEK293T cells were transfected using GeneJuice Transfection Reagent (10 µg DNA/10 cm dish). 48–72 h after transfection, cells were lysed in lysis buffer (50 mM HEPES, 150 mM NaCl, 10% glycerol, 1 mM EGTA, 1 mM EDTA, 25 mM NaF, 10 µM ZnCl2 pH 7.5) supplemented with 10 mM NEM to inhibit deubiquitinating enzymes as well as protease and phosphatase inhibitors (10 µg/ml Aprotinin; 2 µg/ml Leupeptin; 1 mM PMSF; 1 mM Na-orthovanadate). Cell lysates were preincubated with 20–25 µl Protein A/G PLUS Agarose (Santa Cruz Biotechnology) to remove unspecifically bound proteins. Immunoprecipitation using anti-GFP antibodies (1.0–1.2 µg; Roche) was performed overnight at 4°C. Proteins bound to GFP-Antibodies were precipitated by adding 25 µl Protein A/G PLUS Agarose (Santa Cruz Biotechnology) followed by incubation for 45 min at 4°C. Precipitates were analysed by western blot analysis as described above considering the following differences. Unspecific binding sites were blocked using TBS (150 mM NaCl, 50 mM Tris, 0.1% Na-Azide, 0.5% v/v phenol red) containing 5% BSA. Primary antibodies were diluted in TBS/5% BSA.

#### Differential centrifugation

Total brain lysates from wt and *ax* ^J^ mice were prepared and homogenized in 1 ml sucrose buffer (320 mM sucrose, 1 mM EGTA, 4 mM HEPES, 1 mM EDTA, pH 7.4, protease inhibitor cocktail (MiniComplete 1 tablet/10 ml Roche). After initial centrifugation at 1,000×g followed by centrifugation at 10,000×g for 10 min, each cell surface membrane-enriched P2 pellet was obtained. P3 pellets mainly contained small organelles and vesicles upon a 1 h centrifugation-step at 100,000×g. Upon an additional centrifugation step at 400,000×g for 1 h, protein complexes were enriched in the P4 pellet [50].

### Surface biotinylation

48 h after transfection, HEK293 cells were incubated (20 min; 4°C) with HEPES containing 1 mM biotinamidohexanoic acid 3-sulfo-N-hydroxysuccinimid-ester sodium salt (Sigma). Remaining biotin reagent was quenched by adding 100 mM glycine (twice for 20 min at 4°C). Cells were washed with ice cold PBS and lysed in PBS containing 1% Triton X-100 and protease inhibitor cocktail (MiniComplete 1 tablet/10 ml Roche). After a 30 min incubation step on ice, followed by a brief centrifugation-step at 1,000×g (5 min, 4°C), 30 µl of the supernatants were loaded on a gel to evaluate the amount of GABA_A_R α1-GFP. After quantification adjusted volumes of supernatants were added to 30 µl of prewashed magnetic Streptavidin MyOne beads (Dynal) to achieve equal amounts of GABA_A_R α1-GFP used for precipitation. Beads were incubated at 4°C for 3 h on a rotation wheel, washed 3 times, collected and boiled in SDS sample buffer.

### Yeast two hybrid system

For protein-protein interaction analysis, the Matchmaker LexA yeast Two-Hybrid system (Clontech, Heidelberg, Germany) was used. Interactions of bait (pGilda) and prey (pJG4-5) fusion proteins were examined by activation of a LEU2 and a lacZ reporter gene [47].

### *In situ* hybridisation

For detection of GABA_A_R α1 mRNAs, antisense oligonucleotides were synthesized encoding the large intracellular loop region between transmembrane domains M3 and M4 (aa 342-356) [51]. In situ hybridization was performed as previously described [51],[52].

## Supporting Information

Figure S1 Immunohistochemical control staining of cerebellar slices derived from (A,C) wt and (B,D) GABA_A_R α1 knockout mice using GABA_A_R α1-specific antibodies (Upstate Biotechnology, New York). A significantly reduced signal in the granular and molecular layer of GABA_A_R α1-deficient cerebella is seen. (C,D) Magnifications of the Purkinje cell layer of (C) wt and (D) GABA_A_R α1 knockout mice. Scale bar: 100 µm. (D) Since GABA_A_R α1-deficient mutants do not carry a complete gene deletion, but express a remaining N-terminal protein fragment that is recognized by the antibody, cells represented by strong GABA_A_R α1 expression levels, such as Purkinje cells and granule cells, show a prominent cytoplasmic staining (white arrowheads) (Schneider Gasser et al. (2007) Eur J Neurosci 25: 3287–3304). Scale bars in (A) and (B): 500 µm. (E,F) Immunocytochemical analysis of Usp14 (green) and (E) synaptic vesicle (SV) protein 2 (blue) or (F) GABA_A_R α1 (red) in cultured cerebellar neurons revealed partial colocalization of Usp14 with both proteins. Scale bar: 5 µm.
(5.27 MB TIF)

Figure S2 Electrophysiological control analysis. (A) No spontaneous inhibitory postsynaptic currents were recorded in the presence of the GABA_A_R antagonist bicuculline in *ax* ^J^ mice (dark line). A slight shift in baseline currents was attributable to the overlap of successive IPSCs. (B) Cumulative amplitude fractions averaged over six cells for *ax* ^J^ and wt mice. Data of the individual cells are shown as dotted lines. (C) Kinetic parameters of rise-times (10–90%) and decay time-constants from 100 individual IPSCs, each. The sIPSCs represented by values below 1.7 ms were analysed separately (somatic origin). A tendency towards slower decay times is indicated through increasing sIPSCs amplitudes and is most apparent at amplitudes above 150 pA in *ax* ^J^ animals, as compared to sIPSCs in wt animals that are in the range of 80 to 150 pA (p = 0.005). Error bars represent SD values.
(0.32 MB TIF)

Figure S3 Activity assay using a HA-tagged ubiquitin vinyl methyl ester (HAub-VME) probe, that covalently modifies active deubiquitinating enzymes with an HA tag [41],[42]. (A) Western blot analysis of protein extracts derived from *ax* ^J^ and wt kidney, as well as from cultured HEK293 cells using HA-specific antibodies, revealed a signal in the height of Usp14 (upper signal) and UCH-37 (lower signal), the latter representing another deubiquitinating enzyme (Holzl et al. (2000) J Cell Biol 150: 119–130). The Usp14 signal intensity (upper) is significantly reduced in *ax* ^J^ kidney extracts, whereas the UCH-37 signal intensities (lower) remain equal, thereby supporting the specificity of the assay. (B) Western blot analysis of the same protein fractions as loaded in (A), using antibodies specific to Usp14 and a proteasomal marker protein Rpn4 (loading control). USP14 is endogenously expressed in HEK293 cells and in kidney tissue derived from wt mice. As expected, Usp14 is not detectable in kidney tissue derived from *ax* ^J^ mutants.
(0.46 MB TIF)

Figure S4 Analysis of GABA_A_R α1 ubiquitination. (A) Immunoprecipitation of GFP-GABA_A_R α1 using anti-GFP antibodies upon HEK293T cell expression of GABA_A_R α1-GFP, GABA_A_R β3, HA-tagged ubiquitin and either Usp14 wt or catalytic mutant, respectively. GFP-GABA_A_R α1 expression was analysed using GABA_A_R α1-specific antibodies (lower panel). Upon membrane stripping and detection with anti-HA antibodies, ubiquitinated forms of GFP-GABA_A_R α1 are visible (upper panel, large bracket right). Note, the signal in the height of app. 75 kDa is due to a protein that unspecifically binds to agarose beads. (B) The marked region (asterisk, small bracket) detected in (A) (70–100 kDa, middle and right lane) is enlarged. Note, more intense blurred signals in the presence of the Flag-tagged Usp14 catalytic mutant, indicative for ubiquitin-conjugated polypeptides, are visible (arrows, left lane), although the expression level of GABA_A_R α1 is lower (Figure S4C, lower panel, right lane). (C) Western blot analysis of total HEK293T cell lysates using anti-Flag (upper panel) or anti-GABA_A_R α1 (lower panel) antibodies.
(1.26 MB TIF)
