# Supplementary figures and images for: The Ataxia (ax J) Mutation Causes Abnormal GABAA Receptor Turnover in Mice
Source: PLoS Genet. 2009 Sep 4;5(9):e1000631. doi: 10.1371/journal.pgen.1000631 (PMC2744266; doi:10.1371/journal.pgen.1000631)

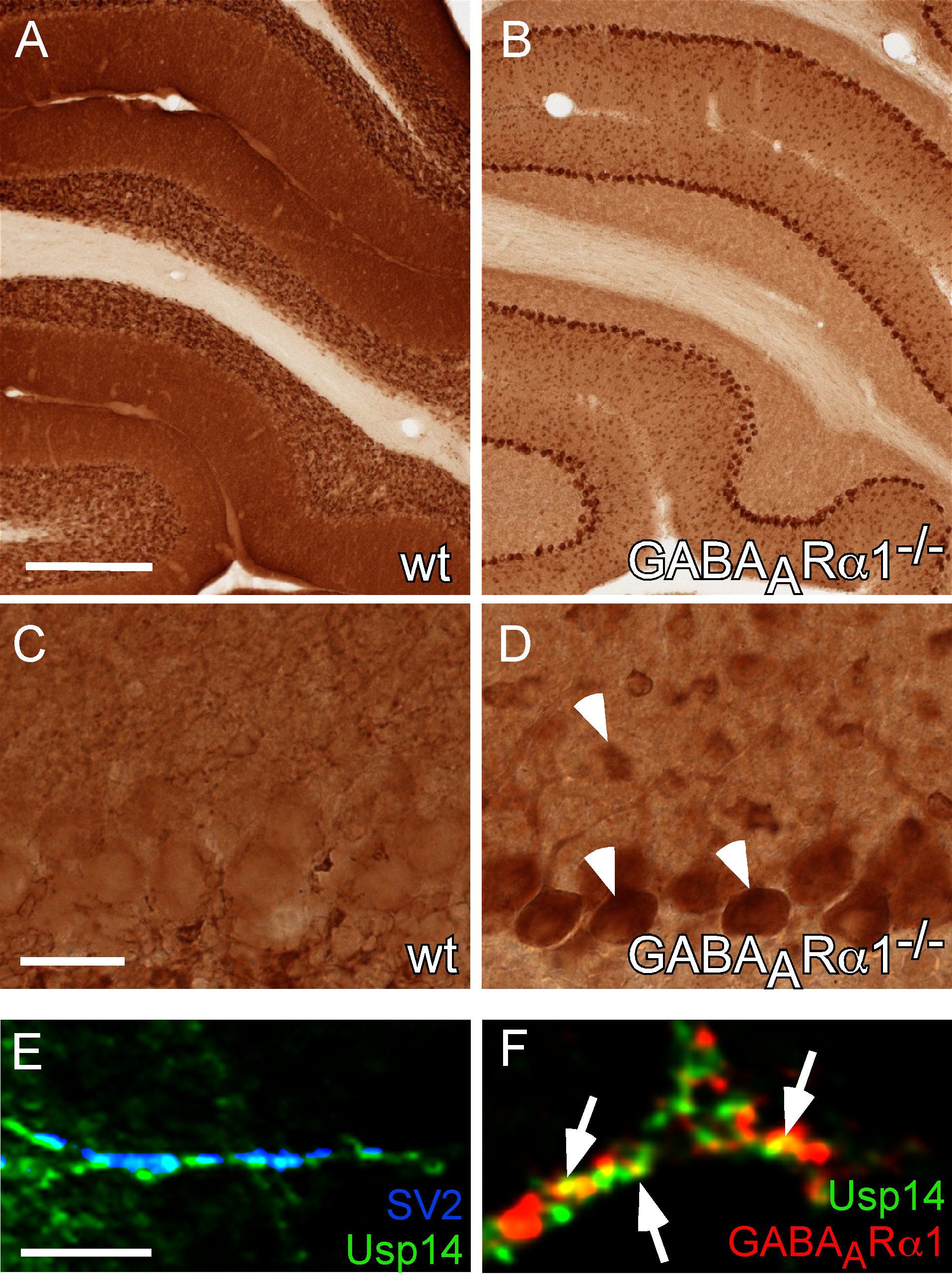

Supplement: Figure S1 — Immunohistochemical control staining of cerebellar slices derived from (A,C) wt and (B,D) GABAAR α1 knockout mice using GABAAR α1-specific antibodies (Upstate Biotechnology, New York). A significantly reduced signal in the granular and molecular layer of GABAAR α1-deficient cerebella is seen. (C,D) Magnifications of the Purkinje cell layer of (C) wt and (D) GABAAR α1 knockout mice. Scale bar: 100 µm. (D) Since GABAAR α1-deficient mutants do not carry a complete gene deletion, but express a remaining N-terminal protein fragment that is recognized by the antibody, cells represented by strong GABAAR α1 expression levels, such as Purkinje cells and granule cells, show a prominent cytoplasmic staining (white arrowheads) (Schneider Gasser et al. (2007) Eur J Neurosci 25: 3287–3304). Scale bars in (A) and (B): 500 µm. (E,F) Immunocytochemical analysis of Usp14 (green) and (E) synaptic vesicle (SV) protein 2 (blue) or (F) GABAAR α1 (red) in cultured cerebellar neurons revealed partial colocalization of Usp14 with both proteins. Scale bar: 5 µm. (5.27 MB TIF) [file pgen.1000631.s001.tif]

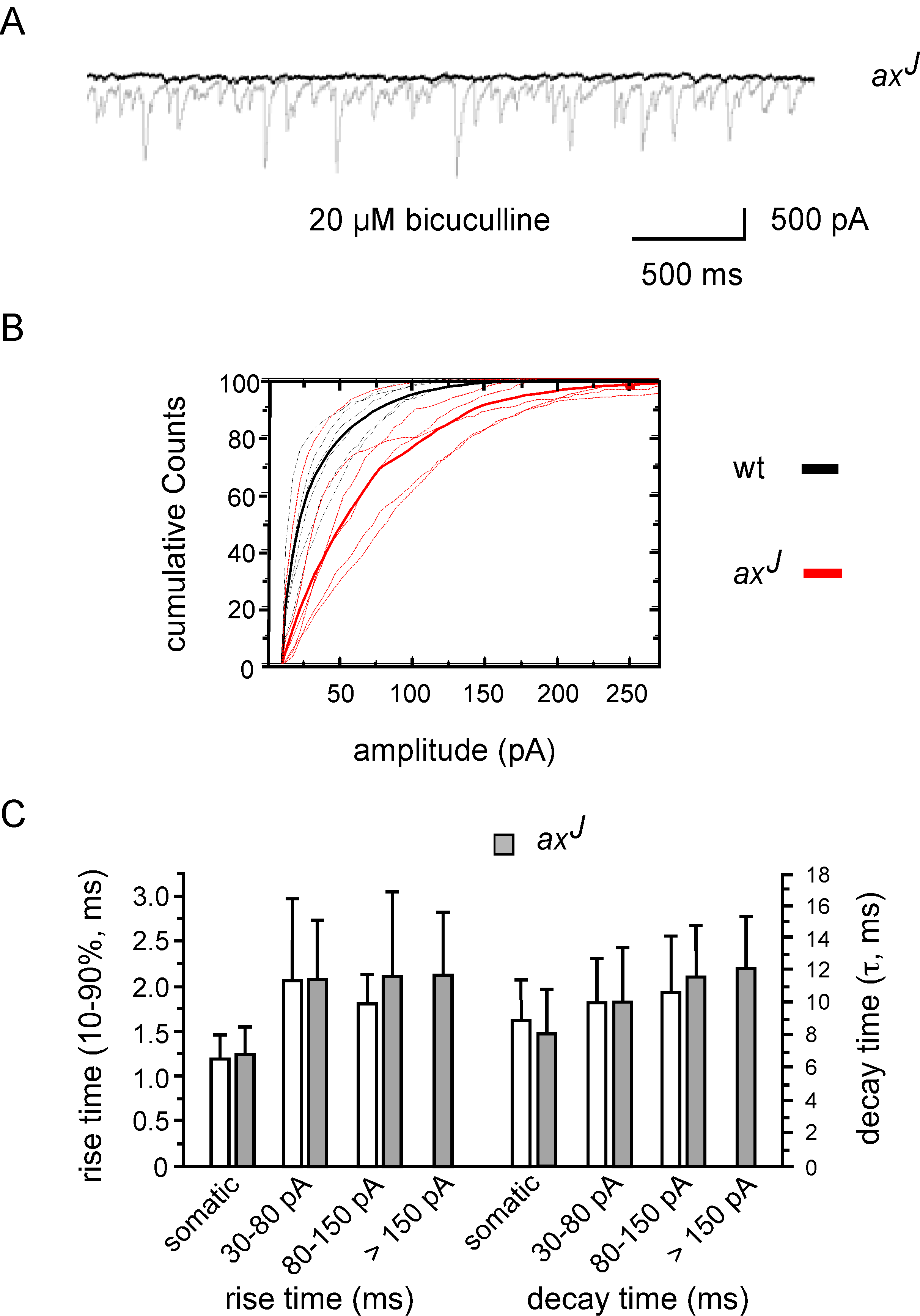

Supplement: Figure S2 — Electrophysiological control analysis. (A) No spontaneous inhibitory postsynaptic currents were recorded in the presence of the GABAAR antagonist bicuculline in ax J mice (dark line). A slight shift in baseline currents was attributable to the overlap of successive IPSCs. (B) Cumulative amplitude fractions averaged over six cells for ax J and wt mice. Data of the individual cells are shown as dotted lines. (C) Kinetic parameters of rise-times (10–90%) and decay time-constants from 100 individual IPSCs, each. The sIPSCs represented by values below 1.7 ms were analysed separately (somatic origin). A tendency towards slower decay times is indicated through increasing sIPSCs amplitudes and is most apparent at amplitudes above 150 pA in ax J animals, as compared to sIPSCs in wt animals that are in the range of 80 to 150 pA (p = 0.005). Error bars represent SD values. (0.32 MB TIF) [file pgen.1000631.s002.tif]

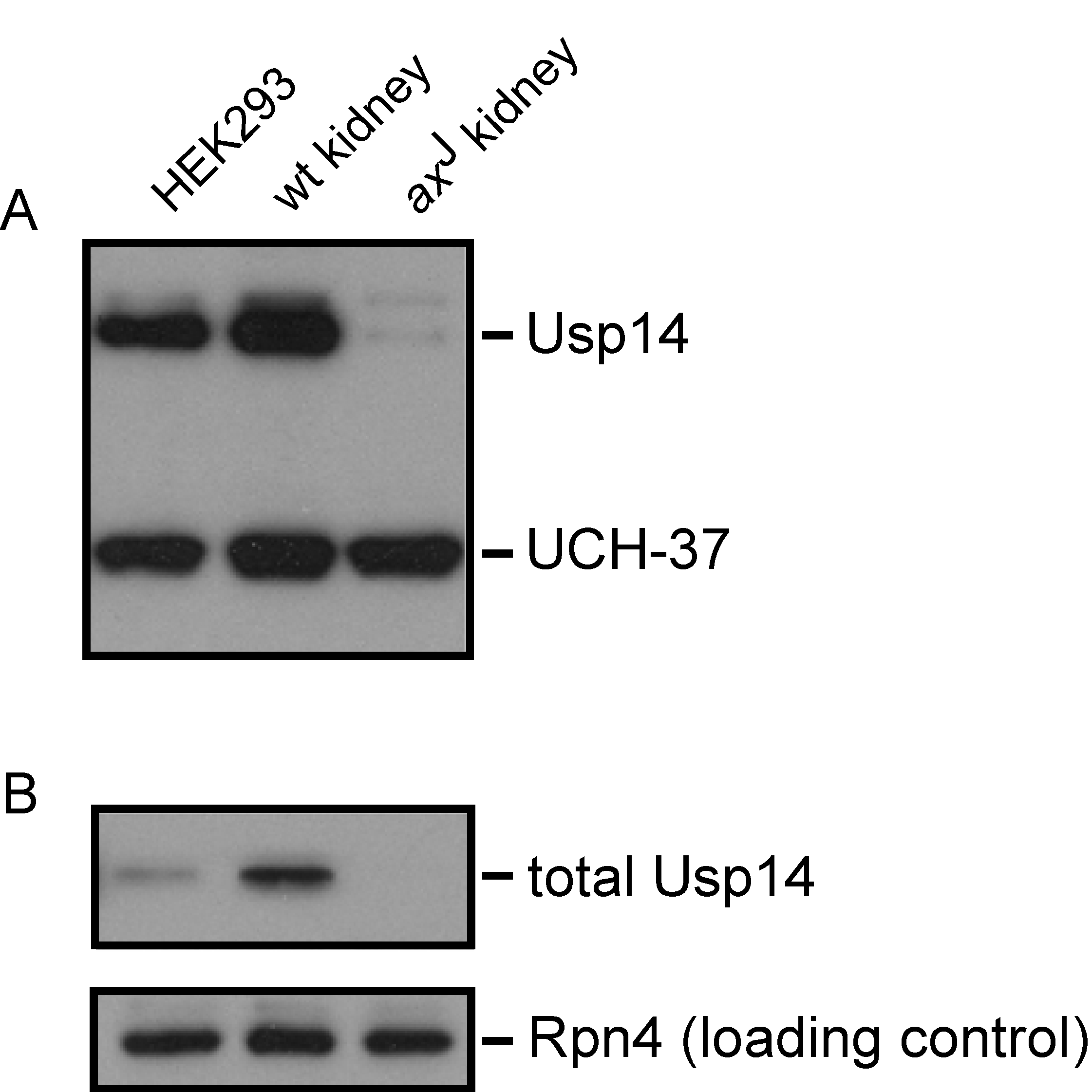

Supplement: Figure S3 — Activity assay using a HA-tagged ubiquitin vinyl methyl ester (HAub-VME) probe, that covalently modifies active deubiquitinating enzymes with an HA tag [41],[42]. (A) Western blot analysis of protein extracts derived from ax J and wt kidney, as well as from cultured HEK293 cells using HA-specific antibodies, revealed a signal in the height of Usp14 (upper signal) and UCH-37 (lower signal), the latter representing another deubiquitinating enzyme (Holzl et al. (2000) J Cell Biol 150: 119–130). The Usp14 signal intensity (upper) is significantly reduced in ax J kidney extracts, whereas the UCH-37 signal intensities (lower) remain equal, thereby supporting the specificity of the assay. (B) Western blot analysis of the same protein fractions as loaded in (A), using antibodies specific to Usp14 and a proteasomal marker protein Rpn4 (loading control). USP14 is endogenously expressed in HEK293 cells and in kidney tissue derived from wt mice. As expected, Usp14 is not detectable in kidney tissue derived from ax J mutants. (0.46 MB TIF) [file pgen.1000631.s003.tif]

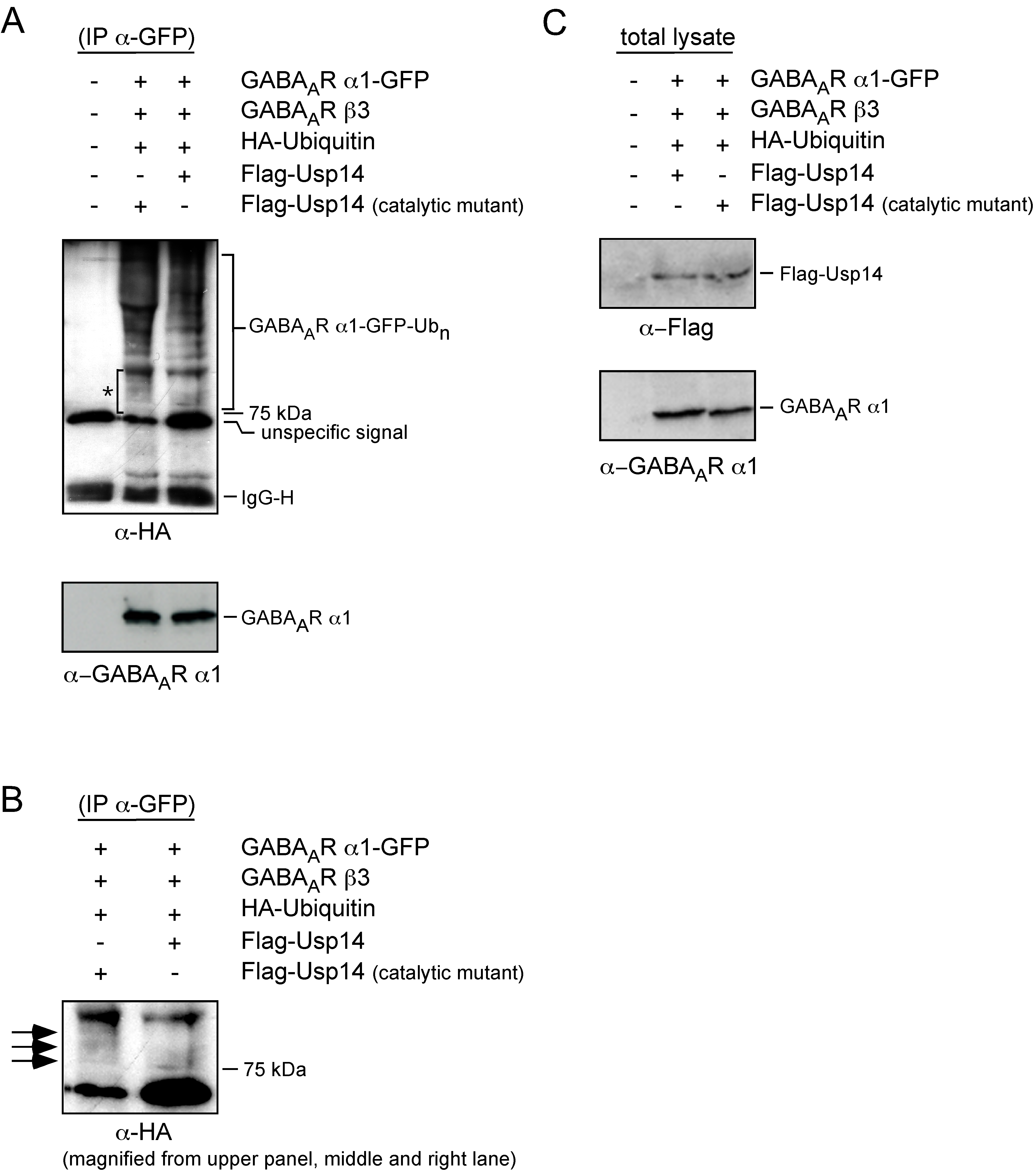

Supplement: Figure S4 — Analysis of GABAAR α1 ubiquitination. (A) Immunoprecipitation of GFP-GABAAR α1 using anti-GFP antibodies upon HEK293T cell expression of GABAAR α1-GFP, GABAAR β3, HA-tagged ubiquitin and either Usp14 wt or catalytic mutant, respectively. GFP-GABAAR α1 expression was analysed using GABAAR α1-specific antibodies (lower panel). Upon membrane stripping and detection with anti-HA antibodies, ubiquitinated forms of GFP-GABAAR α1 are visible (upper panel, large bracket right). Note, the signal in the height of app. 75 kDa is due to a protein that unspecifically binds to agarose beads. (B) The marked region (asterisk, small bracket) detected in (A) (70–100 kDa, middle and right lane) is enlarged. Note, more intense blurred signals in the presence of the Flag-tagged Usp14 catalytic mutant, indicative for ubiquitin-conjugated polypeptides, are visible (arrows, left lane), although the expression level of GABAAR α1 is lower (Figure S4C, lower panel, right lane). (C) Western blot analysis of total HEK293T cell lysates using anti-Flag (upper panel) or anti-GABAAR α1 (lower panel) antibodies. (1.26 MB TIF) [file pgen.1000631.s004.tif]
